# Structural basis for inhibition of the cardiac sodium channel by the atypical antiarrhythmic drug ranolazine

**DOI:** 10.1038/s44161-023-00271-5

**Published:** 2023-05-04

**Authors:** Michael Lenaeus, Tamer M. Gamal El-Din, Lige Tonggu, Ning Zheng, William A. Catterall

**Affiliations:** 1 Division of General Internal Medicine, Department of Medicine, University of Washington, Seattle, WA, USA.; 2 Department of Pharmacology, University of Washington, Seattle, WA, USA.; 3 Howard Hughes Medical Institute, University of Washington, Seattle, WA, USA.

Voltage-gated sodium (Na_V_) channels generate the upstroke of the cardiac action potential by activating rapidly in response to depolarization and conducting Na^+^ inward across the membrane^1,2^. Na_V_1.5 is the predominant Na_V_ channel in the heart^3,4^. It is the molecular target for class I antiarrhythmic drugs (AADs), which often have unwanted side effects, including arrhythmias^5,6^. In contrast, the atypical AAD ranolazine is effective in treatment of atrial arrhythmias and angina pectoris, but with less proarrhythmia than traditional AADs^7–11^. Structures of Na_V_ channels from prokaryotes^12^, skeletal muscle^13^, nerve^14^ and heart^15^ have been determined with AADs bound within the pore to physically block Na^+^ conductance^12,15–19^. Here we use electrophysiology and cryogenic electron microscopy to define the interaction of ranolazine with Na_V_1.5 at high resolution. We reveal ranolazine’s binding pose and elucidate distinct molecular interactions that might underlie its mechanism of action and high therapeutic index relative to traditional class I AADs.

Voltage-gated sodium channels in eukaryotes consist of tissue-specific high-molecular-weight pore-forming α-subunits of ~2,000 amino acid residues that contain four homologous domains (DI–DIV)^20–22^. Each domain of the α-subunit is composed of six transmembrane helices organized in two functional modules: S1–S4 form the voltage-sensing module (VS), whereas S5, S6 and the P loop between them form the pore module (PM). The VS and PM are connected by the α-helical S4–S5 linker. The intracellular linker connecting DIII and DIV serves as the fast inactivation gate, which closes the pore within 1–2 ms after opening^18,23^.

The class ID antiarrhythmic drug (AAD) ranolazine blocks the cardiac voltage-gated sodium channel Na_V_1.5 and is used to treat cardiac arrhythmias and angina pectoris^24–26^. Recent clinical trials show that it is effective in treatment of the increasingly common arrhythmia atrial fibrillation, both alone and in combination with the class III AADs dronedarone and amiodarone^9,10^. Its mechanism of action is distinct from traditional AADs in that it preferentially blocks the late sodium current (*I*_NaL_), a small, slowly decaying component of sodium current that is increased in magnitude by genetic mutations, myocardial ischemia, congestive heart failure and other cardiac diseases^10,27^. The effect of ranolazine on *I*_NaL_ is approximately 10–40-fold greater than its effect on peak sodium current, even though these currents are both conducted by Na_V_1.5 (ref. 28). Understanding the structural basis for the unique pharmacological actions of ranolazine would give fresh insight into the biophysical basis for *I*_NaL_ and provide a template for development of structurally related AADs of greater potency and efficacy.

Ranolazine’s structure contains a substituted dimethylbenzyl ring, similar to the class IB AAD lidocaine, which is fused through a long chain and ether linkage to a second aromatic methoxyphenoxyl ring (Fig. 1a). In this Letter, we tested ranolazine block of a truncated rat construct, rNa_V_1.5c (ref. 15), using whole-cell voltage clamp applied to human embryonic kidney 293 (HEK293) cells. Ranolazine reduced peak current of rNa_V_1.5c in a concentration-dependent manner (Fig. 1b). Its level of inhibition increased with repetitive pulsing, exhibiting the use dependence that is characteristic of sodium-channel-blocking AADs. The dose–response curve for ranolazine blockade reveals an IC_50_ of 110 μM when depolarizing pulses are applied at 0.2 Hz (Fig. 1b), similar to that measured for human Na_V_1.5 in ventricular myocytes^28^. The half-maximal inhibitory concentration (IC_50_) for ranolazine block of rNa_V_1.5c decreased to 80 μM with application of 1 Hz repetitive pulses. Ranolazine reduces maximum sodium conductance and shifts the midpoint of the conductance/voltage curve to more negative membrane potentials (Fig. 1d).

We overexpressed and purified rNa_V_1.5c (ref. 15) in HEK293 cells in the presence of ranolazine and its intracellular binding partners fibroblast growth-factor homology factor 12B (FGF12B) and calmodulin^29^. The resulting complex eluted as a single homogeneous peak on size exclusion chromatography (SEC) and was deposited on grids for structural analysis by cryogenic electron microscopy (cryo-EM) (Extended Data Fig. 1). A total of 2,154,834 particles were analyzed to yield 165,532 particles used to perform 3D reconstruction of the structure of the ranolazine/Na_V_1.5 complex at a resolution of 3.2 Å (Extended Data Figs. 2 and 3). The structure of rNa_V_1.5c shows the classical features of a voltage-gated sodium channel, with four homologous subunits or domains forming a central PM and four VS located in approximately symmetrical positions on the periphery (Fig. 2a). The cryo-EM density is well fit, and the overall backbone structure is very similar to that for rNa_V_1.5c in complex with flecainide, FHF12B and calmodulin (root mean square deviation (RMSD) 0.89 Å; Fig. 2a and Extended Data Tables 1 and 2)^15^.

Ranolazine blocks rNa_V_1.5c by binding in the central cavity of the PM, physically occluding it and blocking ion permeation (Fig. 2b,c). The bound drug is well resolved in our cryo-EM maps (Fig. 2d). The ranolazine molecule is positioned asymmetrically in the central cavity of rNa_V_1.5c through formation of close contacts with the S6 segments of DI and DIV (Fig. 2b,c) and more distant contacts with DIII. Ranolazine is located directly on the intracellular side of the ion selectivity filter, ideally positioned to occlude the ion permeation pathway with its positive charge, while its hydrophobic regions interact with the nearby S6 segments of the PM (Fig. 2b,c).

Ranolazine binds the traditional AAD receptor site in DIV-S6 by making a T-shaped π–π interaction (π-teeing) between its lidocaine-like aromatic ring and the side chain of F1762 (4.2 Å, Fig. 2e), the canonical residue shown to bind the aromatic ring of local anesthetics and AADs^30,31^. Consistent with direct block of the ion permeation pathway, ranolazine also makes contact with small polar residues on the intracellular side of the ion selectivity filter (S1712) and approaches the highly conserved lysine (K1421) known to be critical for sodium selectivity (Fig. 2e). Ranolazine also makes contacts with residues on DI-S6 by approaching Q372 on the intracellular side of DI pore helix and making a T-shaped π–π interaction between the aromatic ring of its atypical methoxyphenoxyl group and F403 (Fig. 2e). F403 is probably a key residue for drug binding based on its position between the methoxyphenoxyl aromatic moiety of ranolazine and its classical class IB AAD core. F403 is also uniquely poised to distribute the effects of drug binding to the pore helix residue Q372 and the flexible, highly conserved gating motif G401/S402 in DI. Ranolazine’s interaction with DI-S6 is consistent with a distinct binding mode that interferes with the gating mechanism of DI-S6, in comparison with class I and III AADs that do not make a similar molecular contact^15,18,19^.

On the basis of these results, we performed mutagenesis and electrophysiological analysis under voltage clamp to explore the role of the identified sites for ranolazine interaction in DI and confirm those previously identified in DIV. We made mutations at the traditional AAD receptor site in DIV (L1464, F1762 and Y1769) and the atypical DI site (Q372 and F403), as well as at one more distant residue in DII (N930) (Fig. 3). The results show greatly decreased use-dependent block for mutations at each site in DI and DIV, with a minimal effect in DII (Fig. 3a). DI and DIV residues also contribute substantially to tonic drug block (Fig. 3b). The effects of the F1762A mutation in DIV on ranolazine binding and block were previously studied in human Na_V_1.5, with similar effects to those shown here^28^, and its key role in drug binding is clear in the structure shown in Fig. 2. L1464 and Y1769 in DIV are further from ranolazine in our structure, and we hypothesize that they influence the secondary structure of DIV-S6 (see below) and the size and shape of nearby fenestrations, which would impede drug exit from its binding site and thereby prolong drug binding, as previously shown for other nearby amino acid residues^30^. Mutations at the atypical Q372/F403 interaction site in DI reduced both tonic and use-dependent blockade by ranolazine by approximately five- to ten-fold in a concentration-dependent manner (Fig. 3 and Extended Data Fig. 4). Neither of these DI residues is part of the classical AAD receptor; therefore, interactions with these residues may be specific for ranolazine binding, consistent with its distinct mechanism of action relative to other AADs.

In addition to these distinct interactions with amino acid side chains in the AAD receptor site, our structure reveals a bend in the DIV-S6 helix resulting from the adoption of a π conformation for residues 1,765–1,769 (Fig. 4a,b)^32^. The π-helix conformation results in rotational movements of the affected side chains relative to central cavity of rNa_V_1.5c and presents a distinct surface in register to the central cavity, as well as the nearby fast inactivation gate particle (IFM), DIV/DI fenestration and activation gate (Fig. 4b). It is notable that the α–π transition is not observed in our previous structures of rNa_V_1.5c (refs. 15,18), which indicates that they may be induced by ranolazine binding. Similar transitions have recently been observed in the peripheral nerve sodium channel Na_V_1.7 and, in a state-dependent manner, in transient receptor potential channels^33,34^.

Overall, there are three major structural features in the ranolazine binding site (Fig. 4c): a π-teeing interaction with F1762 on a partially unwound, π-helix segment of DI-VS6 (orange), polar and electrostatic interactions with the ion selectivity filter region (raspberry) and hydrophobic interactions with DI-S6 that include π-stacking interactions with F403 (green). Alanine-scanning mutagenesis experiments on Na_V_1.2 first suggested the role of F1762 (or equivalent) in binding local anesthetics, AADs and other sodium-channel-blocking drugs^30,31^. Subsequent experiments have confirmed the role of F1762 in ranolazine binding^28^, and cryo-EM structures of Na_V_1.5 with the AAD’s quinidine and propafenone have shown a π–π interaction with the phenyl ring of F1762, which is expected to greatly stabilize drug binding^18,19^. Our data reveal this binding interaction for ranolazine in atomic detail. Its dimethylbenzyl ring makes a strong π-teeing interaction, which may be shared among AAD and other sodium-channel-blocking drugs, including local anesthetics acting on sodium channels in peripheral nerves and antiepileptic drugs acting on sodium channels in the brain. Comparison of our results with previous structures of Na_V_1.5 reveals a key role for residues V1765 and V1766, which have changed positions with the adoption of the π-helix conformation, placing them in close van der Waals contact with the underside of the dimethylbenzyl ring of ranolazine’s lidocaine moiety (Extended Data Fig. 5a,b). These amino acid residues are important for use-dependent binding of the lidocaine analog etidocaine^30^. Our structure reveals that these residues shift their positions and create π-helix-dependent binding interactions for the dimethylbenzyl ring of ranolazine (Extended Data Fig. 5c, top, dark blue), which are not present in the α-helix conformation (Extended Data Fig. 5c, bottom). The α–π transition may have similar effects on other drugs containing the dimethylbenzene ring (Extended Data Fig. 5d).

Recent high-resolution structures showed an α–π transition for DIV-S6 in Na_V_1.7 channels induced by distant binding of extracellular gating modifier toxins and resulting conformational changes that were transmitted inward to DIII-S6 and DIV-S6 at the same helical bend identified here in the ranolazine structure^33^. Our structure shows that the π conformation of DIV-S6 in Na_V_1.5 can be induced and/or stabilized locally by drug binding. Similar structural changes have been observed for ligand binding to other ion channels^35,36^. The π conformation of DIV-S6 induced in Na_V_1.5 by ranolazine binding suggests this transition may be an important part of the gating cycle for voltage-gated sodium channel^33^, which may be captured by ranolazine binding that traps the IFM motif in its receptor site, as suggested in recent molecular modeling studies^36^.

Our electrophysiology results also show strong effects for mutations at sites L1464 and Y1769, even though these residues make more distant interactions with bound ranolazine. We hypothesize that these residues act allosterically on drug binding by orienting the phenyl ring of F1762 in an optimal position for π–π interactions and thereby influencing the secondary structure of DIV-S6 and its interaction with IFM motif. The bulky side chain of L1464 approaches F1762 from across the DIII/DIV fenestration and probably restricts its motion during the α–π transition, while the side chain of Y1769 undergoes a large rotational movement during α–π transition that decreases the size of the nearby DIV/DI fenestration and is expected to stabilize the ranolazine-bound position of DI residue F403 by causing a clash in the corresponding position identified in a previous structure containing only alpha-helix DIV-S6 (Extended Data Fig. 6a,b). Data showing that the mutation Y1769C potentiates ranolazine inhibition of Na_V_1.5 channels also supports an interaction between Y1769 and ranolazine^37^, even though the inhibiting versus potentiating effects of the alanine and cystine substitutions are distinct.

The binding pose of ranolazine in rNa_V_1.5c is compared with those of the class IA, IB and IC AADs in Fig. 4c–f. The striking π-teeing interaction of ranolazine (Fig. 4c, yellow) overlaps that found for class IA AAD quinidine (Fig. 4d, light blue). Ranolazine and quinidine bind to DIV-S6 in a similar fashion and make an overlapping π–π interaction with F1762 (Fig. 4c,d), highlighting a common mode of AAD interaction through the aromatic rings in AADs and F1762. However, compared with ranolazine, quinidine does not stabilize the π-helical conformation of DIV-S6 and makes only a minor contact with DI (Fig. 4d), as confirmed by only mild effects of mutations of F403 to Ala on quinidine binding^19^. Moreover, ranolazine’s interactions with the intracellular ends of the DIII and DIV pore helices and the selectivity filter are also stronger and more extensive than the corresponding interactions with quinidine (Fig. 4d). These results highlight the differential binding contacts of ranolazine compared with the class IA AAD quinidine.

The binding pose for class IB AADs has not yet been determined. However, overlay of the structure of the class IB AAD mexiletine reveals the likely points of interaction that overlap with the lidocaine-like moiety of ranolazine (Fig. 4e). In agreement with this binding pose for class IB AADs, mutagenesis studies of K1421, S1712 and F1762 show clear effects on the binding and action of mexiletine and the quaternary derivative QX-314 (refs. 38–40). This proposed π-helix-mediated mechanism of ranolazine binding may underlie state-dependent binding of class IB AADs and other lidocaine-like drugs, which contain the same dimethylbenzyl ring and also show preferential activity against *I*_NaL_, though to a lesser degree than ranolazine (Extended Data Fig. 5c,d). Further structural experiments are necessary to test this hypothesis for class IB AADs and local anesthetic binding and understand the basis of class IB AAD action at high resolution.

The class IC AAD propafenone binds to Na_V_1.5 in a high-affinity pose with its two aromatic rings reaching toward the walls of the central cavity to interact with F1762 and Y1769 (Fig. 4f)^18^, which form high-affinity interactions with bound AADs^30,31,41^. Drug binding is stabilized by a π–π interaction of these aromatic side chains with this binding pose of propafenone (4.7 Å) and by on-edge van der Waals interactions with Y1769. Bound propafenone also interacts with Q372 and F403 in the pore helix in DI (Fig. 4f). However, comparisons of the binding poses of ranolazine and propafenone (Fig. 4f) suggest that the extended structure of ranolazine allows it to make distinct π–π interactions with F1762 and more productive interactions with the DI pore helix and selectivity filter, thereby mediating its distinct pharmacological properties.

Ranolazine’s clinically relevant actions occur through high-affinity (~5 μM) block of the late sodium current, *I*_NaL_, which is increased in cardiac disease and in patients carrying genetic mutations that destabilize inactivation^27^. Ranolazine can also can block peak *I*_Na_ in experimental preparations^24^. However, its IC_50_ for inhibition of peak *I*_Na_ is ~10-fold higher than the therapeutic concentration of 2–8 μM in plasma; therefore, inhibition of peak *I*_Na_ by ranolazine is relevant only in the context of pathological depolarization, rapid action potential firing, or both, as in atrial fibrillation^24^. Like class IB AADs, ranolazine binds and dissociates during each cardiac cycle at therapeutic concentration, which prevents cumulative inhibition of the peak sodium current. The minimal effect of ranolazine on peak *I*_Na_ at therapeutic concentrations, paired with its high affinity for late *I*_Na,_ probably explains its lack of proarrhythmia relative to other class I AADs, which exert their sodium-channel blocking effects primarily on peak *I*_Na_ and cause substantial cumulative block over time^26^. The structural interactions presented here provide a compelling explanation for these differential effects compared with class I AADs by showing that ranolazine acts as a structural clamp that induces tight binding of the IFM inactivation particle to the π-helical form of DIV-S6 and thereby prevents the reopenings that generate *I*_NaL_. We hypothesize that the unstable inactivation gating that produces *I*_NaL_ releases DIV-S6 from the IFM inactivation particle during the repolarization phase of the gating cycle, thereby allowing increased availability of the π-helical form of DIV-S6 for drug binding. In this context, ranolazine’s unique chemistry and binding kinetics allow it to restore inactivation to these channels by capturing the π-helix (Fig. 4a,b), both during drug binding and for tens of milliseconds after drug dissociation, which allows peak sodium current to flow and prevents activation of *I*_NaL._

Understanding the structural basis for binding and block of Na_V_1.5 by the different classes of AADs is important because these drugs have distinct clinical uses, as well as drug-specific unwanted side effects that are often life threatening, as demonstrated by the CAST trial^42^. Our overall hypothesis for specific binding and block of Na_V_1.5 by class IA, IB and IC AADs is that they share a core binding region, as defined in previous structure–function studies, but also reach out to interact with other neighboring regions of the receptor region in a drug-specific manner. The results presented here provide strong support for this hypothesis by showing that ranolazine interacts with the traditional AAD receptor in a similar fashion to class IA, IB and IC AADs but makes distinct contacts by interacting with a π-helix segment of DIV-S6, as well as with the pore helices, selectivity filter and DI. These structural results provide a molecular template for design and development of next-generation AADs with greater efficacy, specificity and safety.

## Methods

### Electrophysiology

All experiments were performed at room temperature (21–24 °C). For measuring the activation curves of Na_V_1.5c-WT and mutant constructs, HEK293S GnTI^−^ cells were held at −140 mV and 50 ms pulses were applied in 10 mV increments. Holding potential (HP) was set to −140 mV. Capacitance was subtracted and series resistance was compensated using internal amplifier circuitry; 80–85% of series resistance was compensated. For measurements of use-dependent block of ranolazine, drug was washed in while the channel was held at −140 mV and repetitive 100 ms pulses from HP −140 mV to 0 mV at a frequency of 1 Hz were applied. For tonic block, a pulse protocol from −140 mV up to +30 mV in 10 mV steps was applied at 0.2 Hz before and after washing in ranolazine. The peak current of the measured family traces before and after washing the drug was taken as a measure of tonic block. Extracellular solution contained in millimolar: 140 NaCl, 2 CaCl_2_, 2 MgCl_2_ and 10 HEPES, pH 7.4; intracellular solution: 35 NaCl, 105 CsF, 10 EGTA and 10 HEPES, pH 7.4. Glass electrodes had a resistance of 2–3 MΩ hm. Currents resulting from applied pulses were filtered at 5 kHz with a low-pass Bessel filter, and then digitized at 20 kHz. Voltage commands were generated using Pulse 8.5 software (HEKA) and ITC18 analog-to-digital interface (Instrutech). Conductance–voltage (*G*–*V*) curves were calculated from the corresponding *I*–*V* curves. *G*–*V* relationships, with or without ranolazine, were fit with a Boltzmann function of the form 1/1[1 + exp[*V* − V_1/2_)/*k*]]. *G*–*V* curve of each cell measured in presence of 150 μM ranolazine was normalized to the the *G*–*V* of the same cell before washing in ranolazine. Inhibition curves were fit with a Hill equation with *n*_H_ = 1.0 unless indicated otherwise in the figure legends.

### Protein expression and purification

We used the Bacmam system and HEK293 GnTl^−^ cells (ATCC CRL-3022) in suspension culture to overexpress a truncated form of the rat cardiac sodium channel (rNa_V_1.5c) as previously described^42^. Ranolazine was added to cell culture at 100 μM during overexpression and maintained at that concentration throughout the purification, until the concentration was increased to 500 μM in the stage before cryo-EM grid preparation The protein was extracted by 1.5% (w/v) *n*-dodecyl-β-d-pyranoside (DDM, Anatrace) and 0.3% (w/v) cholesterol hemi-succinate (CHS, Sigma) in Buffer A, containing 25 mM HEPES pH 7.4, 150 mM NaCl, 10% glycerol, protease inhibitors (cOmplete Protease Inhibitor Cocktail, Roche) and 100 μM ranolazine (TargetMol). After centrifugation, the protein was mixed with anti-FLAG M2 resin (Sigma) that was equilibrated with Buffer A including 0.06% glyco-disogenin (GDN, Anatrace) and incubated for 2 h at 4 °C. The protein was subsequent eluted with buffer B, containing 25 mM HEPES pH 7.4, 150 mM NaCl, 0.06% GDN, 100 μM ranolazine and 1 mM FLAG peptide. The resulting elution was concentrated in 100 kDa molecular weight cut-off concentrators, then purified by SEC (Superose 6) using Buffer B without FLAG peptide. Peak fractions were combined, concentrated and subjected to a second run of SEC, using Buffer C (25 mM imidazole pH 6.0, 150 mM NaCl, 0.006% GDN and 500 μM ranolazine). Peak fractions were then combined and concentrated to a final protein concentration of 4.5 mg ml^−1^ (50 μl final volume).

### Cryo-EM sample preparation and data acquisition

Cryo-EM grids were prepared by applying freshly purified protein samples to glow-discharged holey carbon grids (Quantifoil Micro Tools GmbH). Grids were blotted for 3–4 s at 22 °C and 100% humidity and immediately plunge-frozen in liquid ethane cooled by liquid nitrogen using Vitrobot Mark IV (Thermo Fisher Scientific). Cryo-EM data were recorded on a Titan Krios microscope (Thermo Fisher Scientific) operated at 300 kV, equipped with a GIF-quantum energy filter (Gatan) at 20 eV slit width and a K2 Summit direct detector (Gatan). LEGINON^43^ was used for automated data collection. A total of 4,800 movies were collected at a nominal magnification of 130,000× in super-resolution mode resulting in a pixel size of 0.528 Å, with a small defocus range of −0.5 to −2.5 μm. The dose rate on the camera was set to be ~8 counts per physical pixel per second. The total exposure time was 8.6 s (0.2 s per frame), leading to a total accumulate dose of 60 electrons Å^−2^ on the specimen.

### Cryo-EM image processing

Movie frames were aligned and 2× binned to a pixel size of 1.056 Å using MotionCor2 (ref. 44). Each frame in the image stack was divided into 5 × 5 patches for anisotropic image motion correction, and dose weighting was carried out to calculate the motion-corrected image. Contrast transfer function estimation was done using GCTF^45^. Subsequent image processing steps were performed using RELION^46^ and CryoSPARC^47^ on an NVIDIA graphics processing unit-accelerated workstation. Particles were autopicked using templates derived from 2D class averages of rNa_V_1.5c wild type using RELION. A total of 2,154,834 autopicked particles were subjected to two rounds of 2D classification to remove bad particles using RELION. A total of 921,237 good particles selected were then subjected to Global3D classification and Local3D classification using RELION. Global3D classification was carried out in RELION using the cryo-EM map of rNa_V_1.5c wild type (PDB 6UZ3) that had been low-pass filtered to 20 Å as an initial template, and three classes were generated. After global angular search, the particles were further subjected to cycles of local angular search and four classes were generated. The best class had 242,182 good particles and resulted in 3.38 A resolution. The 242,182 good particles stack was then imported into cryoSPARC and subjected to hetero refinement and non-uniform refinement. A total of 165,532 particles were used for the final reconstruction. The final map was reported at 3.20 Å according to gold-standard Fourier shell correction criterion.

## Extended Data

**Extended Data Fig. 1 |:**
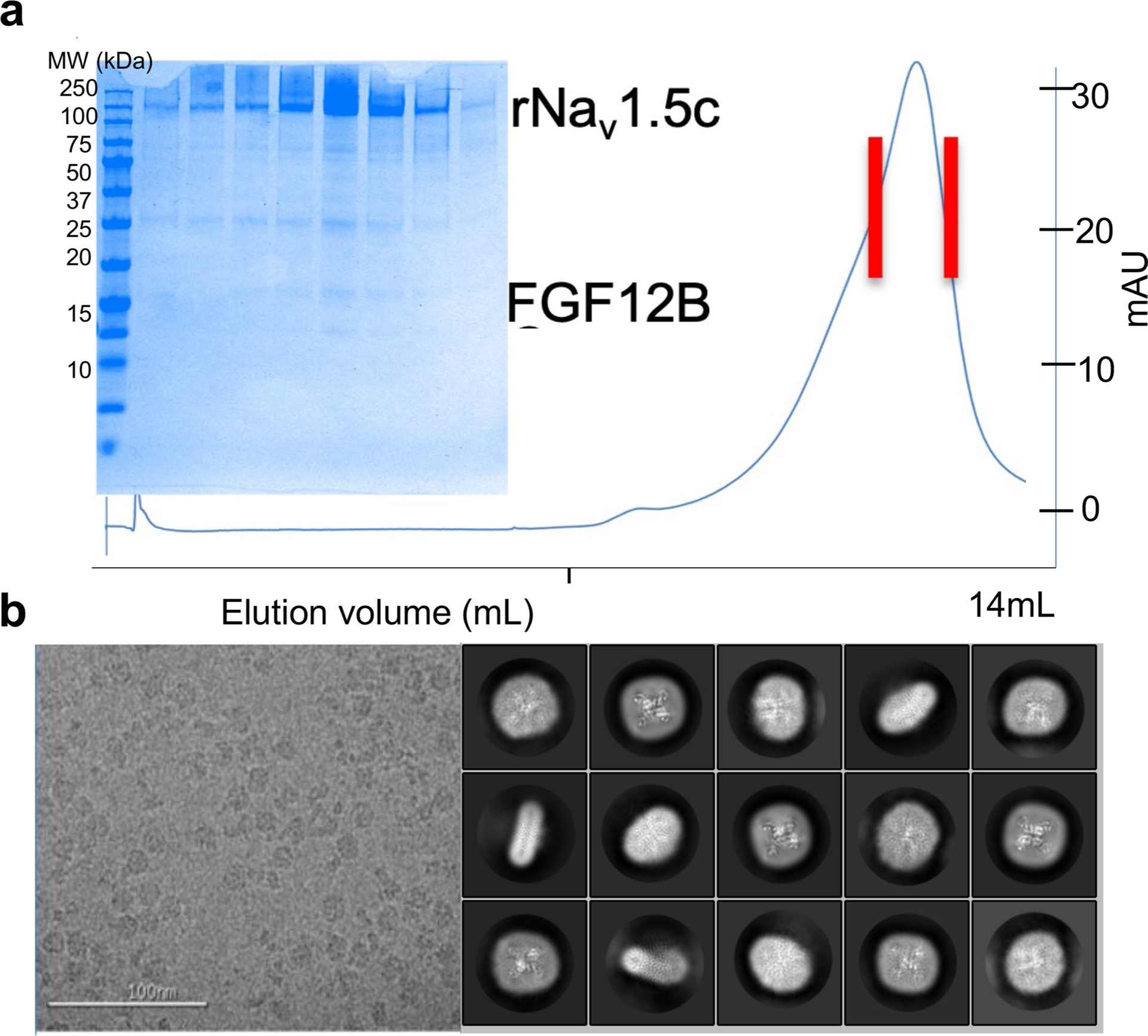
The purification of rNa_v_1.5c and preparation of samples for cryo-EM. **a,** The size-exclusion chromatography profile is shown for the final step of purification of the rNav1.5c, FGF12B, calmodulin and ranolazine complex with red lines indicating the sample moved forward for cryo-EM analysis. This experiment was only performed once. **b**, Sample micrograph collected during cryo-EM data collection and representative 2D classifications as determined by Relion. Information on cryo-EM data collection and data processing is shown in Extended Data Fig. 2.

**Extended Data Fig. 2 |:**
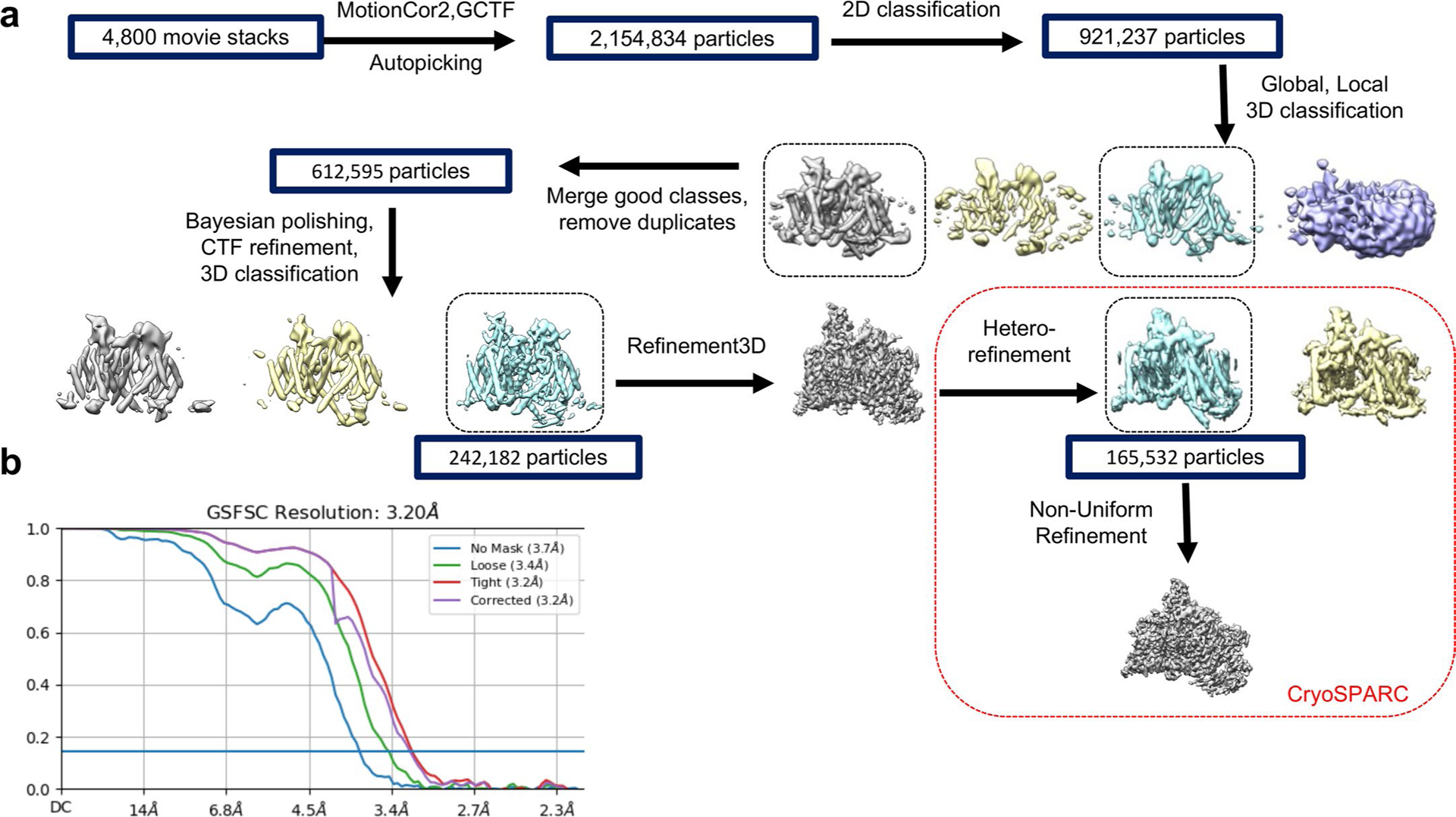
Cryo-EM data processing and 3D reconstruction. **a**,Flowchart for EM data processing. **b**, Gold-standard FSC curve for the 3D reconstruction of rNa_v_1.5c, FGF12B, calmodulin and ranolazine complex.

**Extended Data Fig. 3 |:**
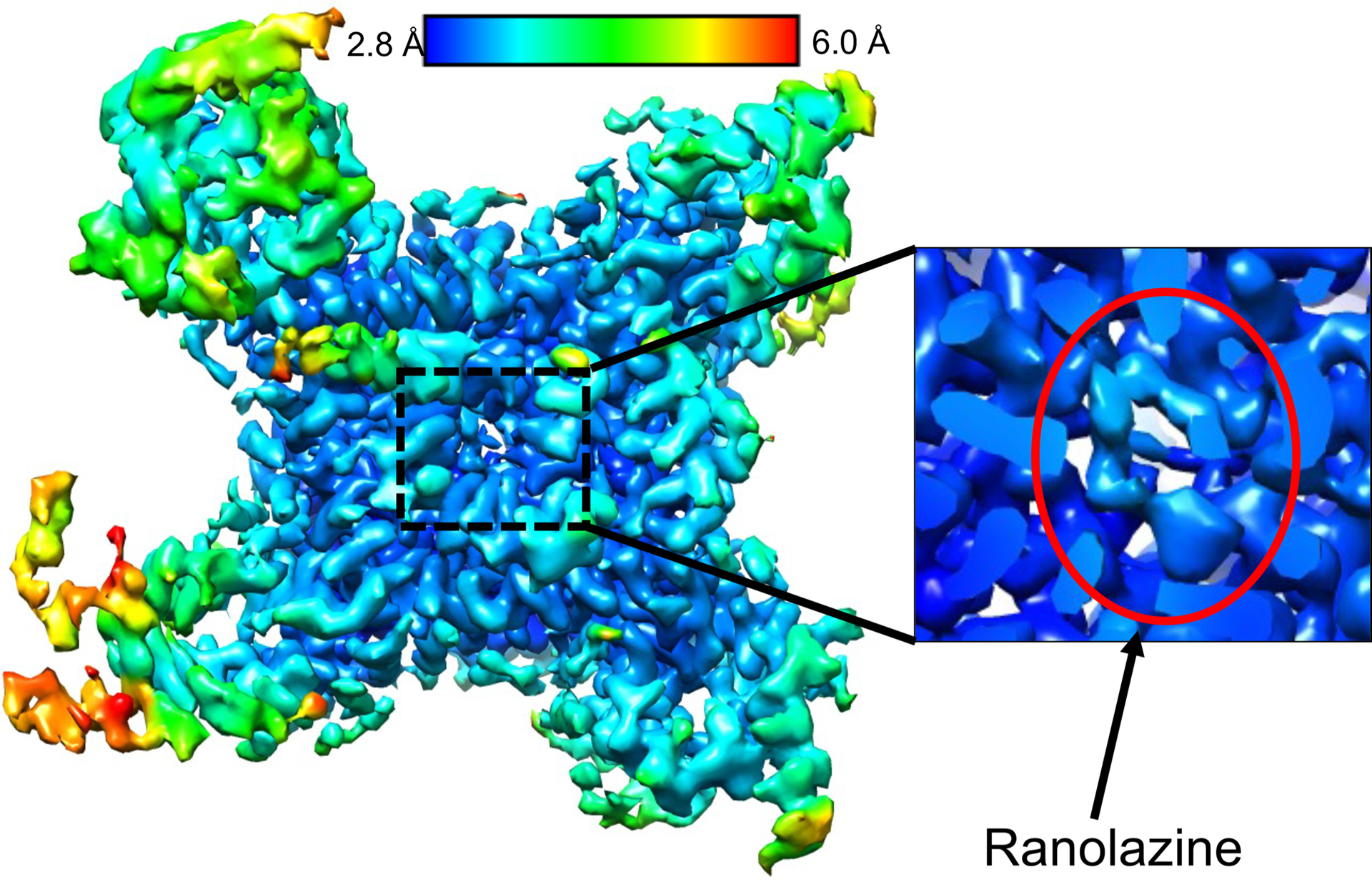
3D reconstruction of rNa_v_1.5c, FGF12B, calmodulin, and ranolazine complex color coded for map resolution. EM density is shown from below rNav1.5c as if one is inside the cell and looking outward at the plasma membrane. The map is colored by resolution with scale shown (blue = high resolution, red = lower). A close-up of the ranolazine site is shown, highlighting the quality of EM density at ranolazine’s binding site. A red circle is shown to identify the EM density corresponding to ranolazine.

**Extended Data Fig. 4 |:**
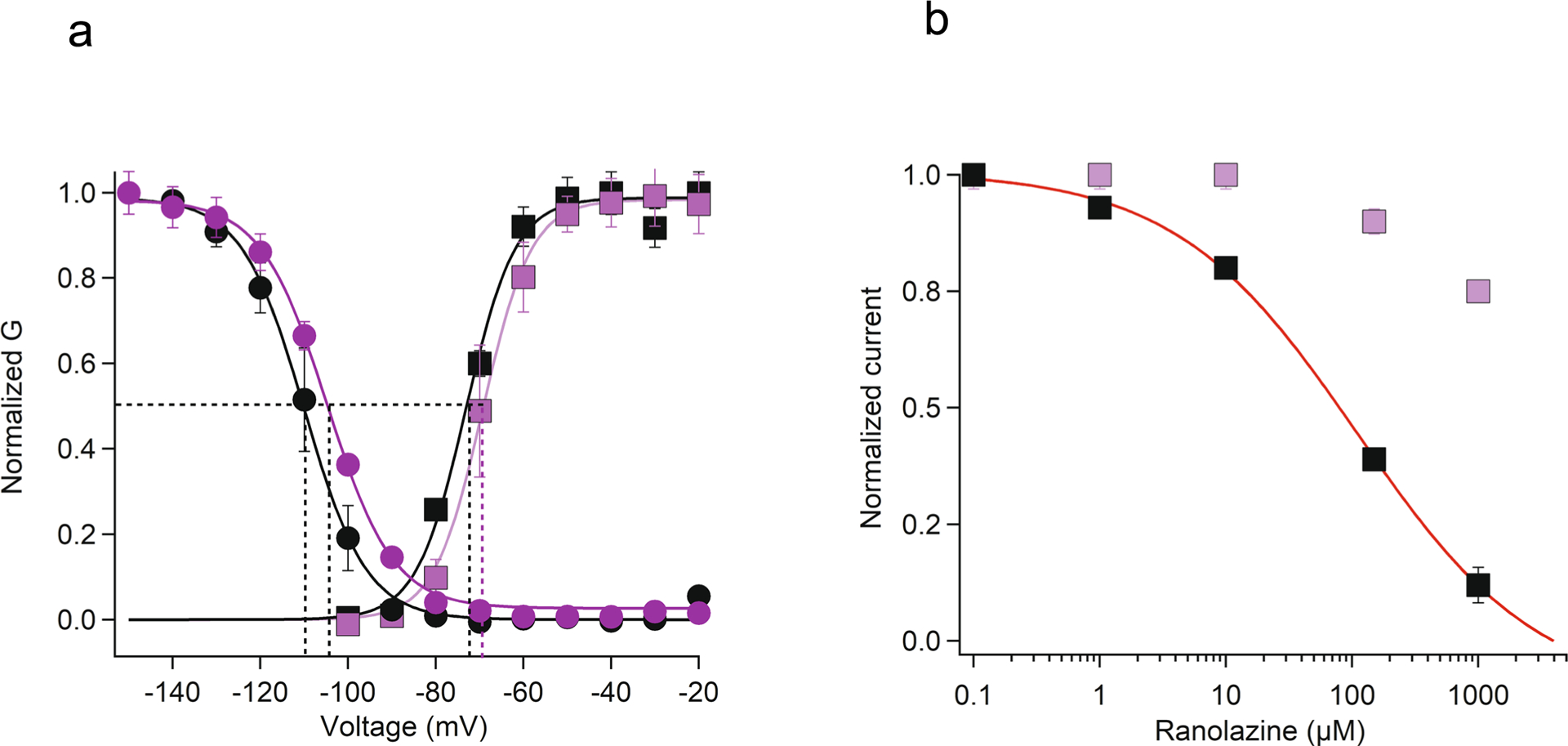
Functional characterization of the Na_v_1.5c mutant Q372A and its effects on ranolazine binding. a. G-V curves of Nav1.5c WT and Q372A mutant derived from I-V relationships. The voltages for half maximal activation and slopes are: WT V_1/2_ = −73 ± 0.8 mV, *K* = 5.6 ± 0.6, Q372A V_1/2_ =−69 ± 0.3 mV, *K* = 5.5 ± 0.3. Steady-state inactivation of Nav1.5c WT and Q372A mutant. Two pulses were applied: a 500-ms conditioning pulse to the indicated potentials followed by 50 ms test pulse to 0 mV. Nav1.5c WT V_h_ = −110 ± 0.4, *K* = 7.8 ± 0.5, Q372A V_h_ = −104 ± 0.6, *K* = 7.6 ± 0.5. WT activation and steady-state inactivation curves N = 4 distinct cells, Q372A GV and SSI curves N = 4 distinct cells. b. Dose-response curve for the tonic block of Q372A compared to Nav1.5c WT. Each concentration is an average of three different cells and each cell is used only once for each concentration. Error bars indicates s.e.m.

**Extended Data Fig. 5 |:**
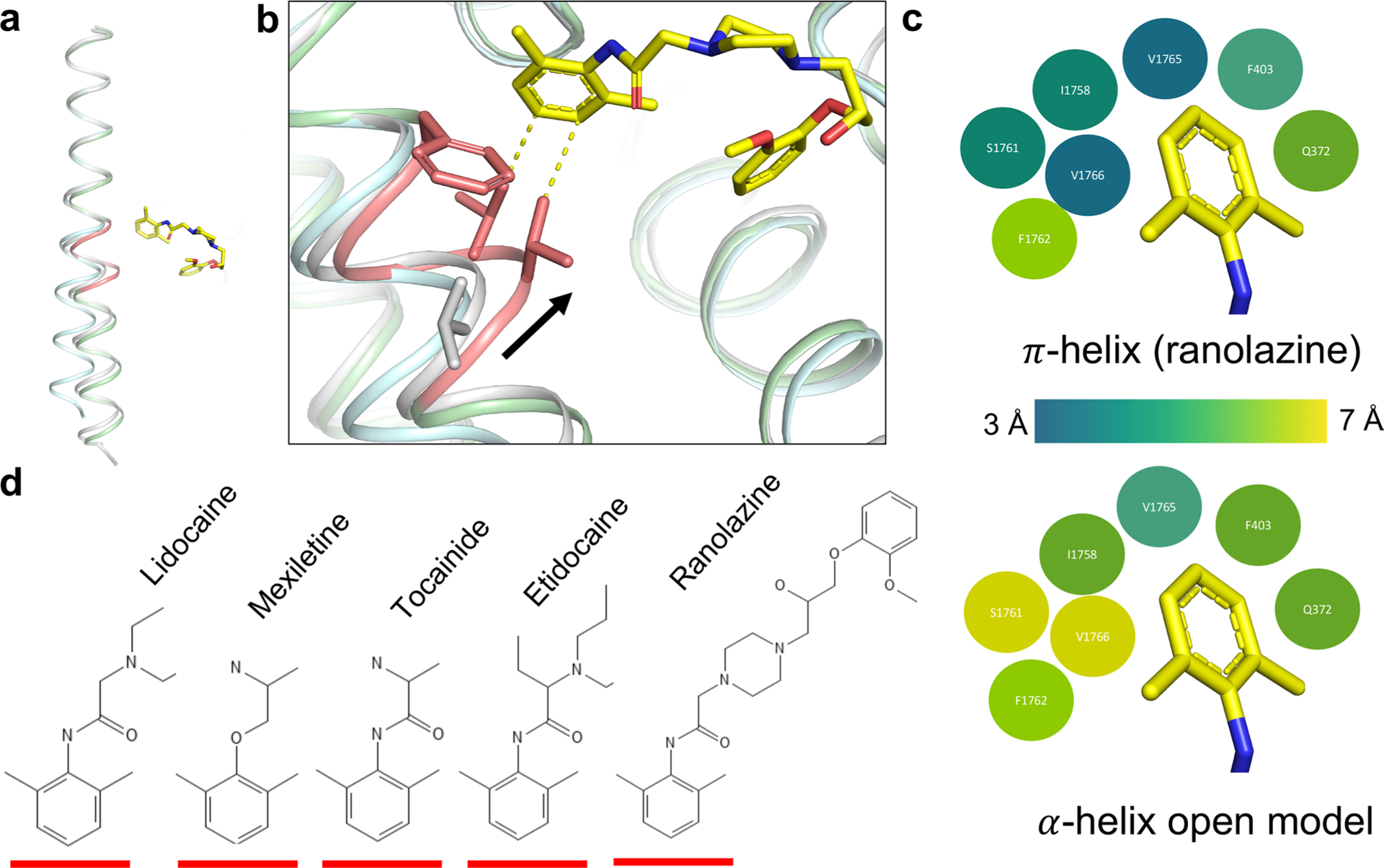
α-π transition of DIV S6 and π-helix dependent binding of ranolazine. **a**, An overlay of DIV-S6 helices from the current structure (light green with pink π-helix highlighted), the apo structure of rNa_v_1.5c (pdb 6uz3, grey), and the open structure of rNa_v_1.5c (pdb 7fbs, cyan). The π-helical portion of DIV-S6 (pink) creates additional drug binding surface in the central cavity of rNa_v_1.5c. **b**, A close-up of the ranolazine binding site with sticks shown for important residues F1762, V1765, and V1766. Coloring is as in part a and the side chain position of V1766 is shown for both the apo structure and the ranolazine-bound structure, with a black arrow highlighting the distance between these alpha carbon positions (2.7 Å). Yellow dashed lines show the strengthened van der waals contacts in the π-helical form of DIV-S6 found in the ranolazine structure (distance is 3.3 Å). **c**, A bubble diagram showing the local differences between π-helical (top) and α-helical (bottom) forms of DIV-S6. Residues are shown as bubbles with coloring dependent on the nearest approach of the side chain to the bound drug according to the color scale shown in the figure. The structure and the dimethylbenzyl moiety of ranolazine is shown for reference. **d**, Stick diagrams of ranolazine and the class IB AADs lidocaine, mexilitine, tocainide, and etidocaine are shown with a red line highlighting each molecule’s dimethylbenzyl moiety.

**Extended Data Fig. 6 |:**
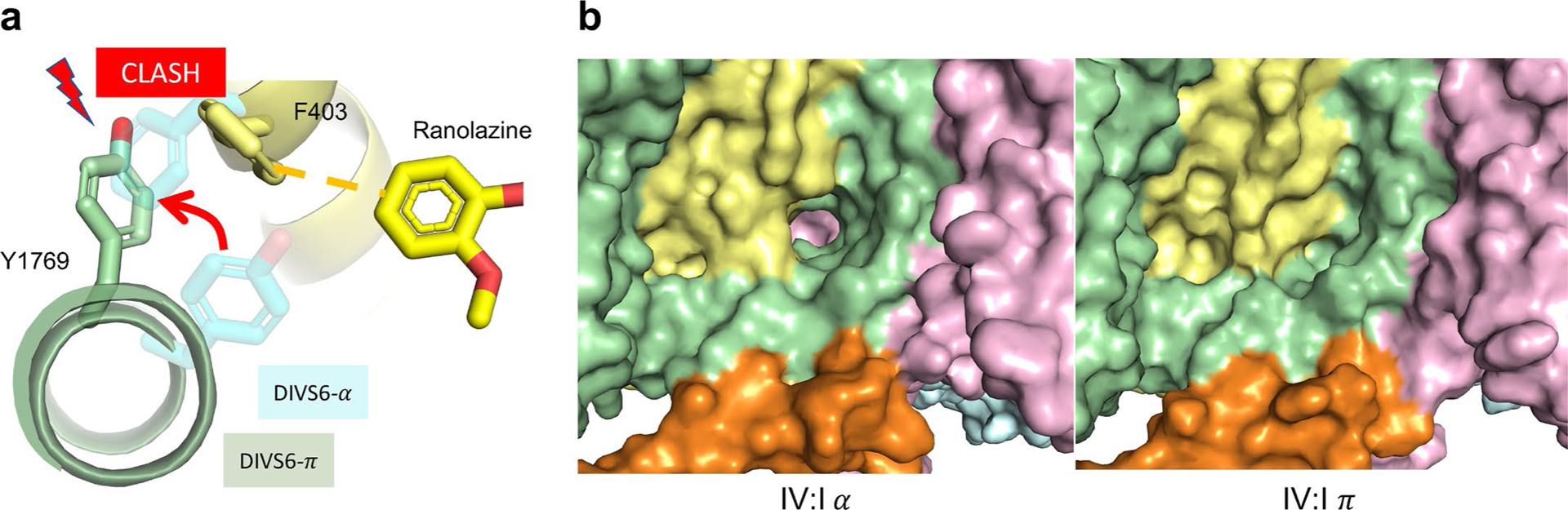
Effect of α-π transition of DIV S6 on the DIV-DI fenestration. **a** Cartoon models of DIV-S6 (green) and DI-S6 (pale yellow) as in earlier figures. Sticks are shown for DIV-S6 residue Y1769 (green, in π helix conformation), F403 (pale yellow) and the non-lidocaine portion of ranolazine that interacts with F403 by π-teeing (dashed line). Transparent sticks are shown in blue for the apo (α-helix) conformations of Y1769 and F403, illustrating the clash between the side chain of π-Y1769 and apo F403. **b**, Surface illustration of DIV/DI fenestration in α and π forms of DIV-S6. Domains are colored as in prior figures - DI pale yellow, DIII, pink, DIV, green, DIII/DIV linker (IFM) orange.

**Extended Data Table 1 |:** Statistical Parameters for cryo-EM data collection

Data Collection and Processing	rNa_v_1.5c, FGF12B, CaM, Ranolazine
	EMD-28887, PDB 8F6P
Magnification	130,000x
Voltage (kV)	300
Electron Exposure (e^−^/Å^2^)	60
Defocus Range	−0.5 to − 2.5 μm
Pixel size (Å)	0.528
Symmetry imposed	C1
Initial particle images	2,154,834
Final particle images	165,532
Map Resolution (Å)	3.20
FSC threshold	0.143

**Extended Data Table 2 |:** Statistical Parameters for Model Refinement

Refinement	rNa_v_1.5c, FGF12B, CaM, Ranolazine
	EMD-28887, PDB 8F6P
Model resolution	3.20 Å
FSC threshold	0.5
Map sharpening B-factor (Å^2^)	−96
Model composition	
Non-hydrogen atoms	9279
Protein residues	1091
Ligand	35
B-factors (Å^2^)	
Protein	56.74
Ligand	58.75
RMSD	
Bonds(Å)	0.003
Angles (°)	0.598
Validation	
Molprobity	1.93
Clash score	16.38
Ramachandran plot (fav/allow/out)	96.65/3.35/0
Poor rotamers	0.12

## Figures and Tables

**Fig. 1 |:**
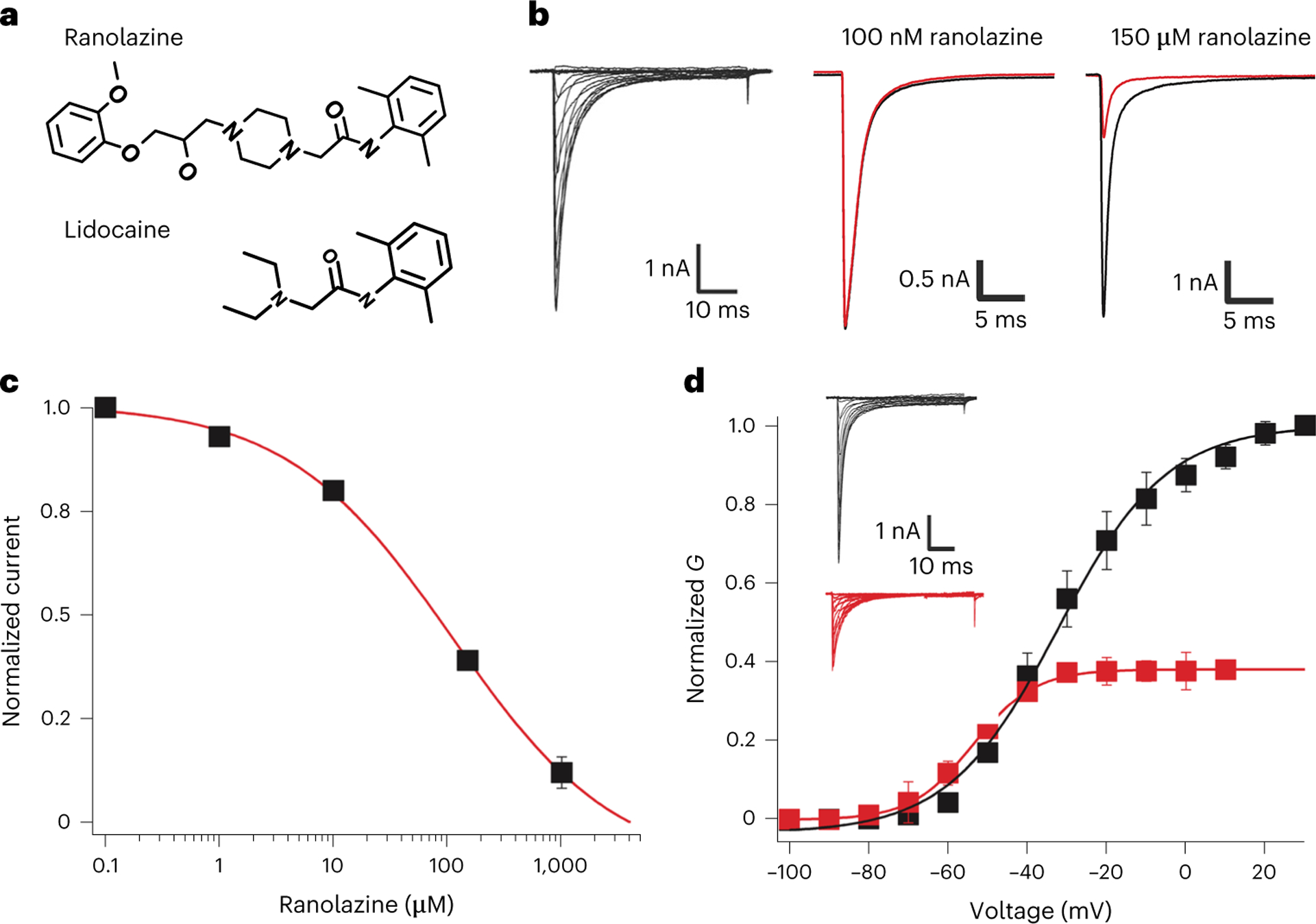
Ranolazine binding and block of rNa_V_1.5c. **a**, The chemical structures of ranolazine and lidocaine, highlighting their similarities and differences. **b**, Sodium current families recorded from rNa_V_1.5c expressed in HEK293 cells (left). Representative current traces recorded during depolarizing steps to 0 mV from a holding potential of −140 mV with drug in the external solution for those experiments containing ranolazine. Drug-free currents are shown in black, and the indicated concentration of ranolazine shown as a red trace. **c**, Dose–response curve for tonic blockade of rNa_V_1.5c by ranolazine. A pulse protocol from −140 mV up to +30 mV in 10 mV steps was applied at 0.2 Hz before and after washing in ranolazine. The peak current of the measured family traces before and after washing the drug was taken as a measure of tonic block. IC_50_, 110 ± 11 μM; Hill coefficient, 0.66. Each concentration is an average of five different cells. Each cell is used only once in these measurements, so that a total of 25 cells were used (5 distinct cells were measured for each concentration of ranolazine studied). **d**, Activation curves of rNa_V_1.5c before (black) and after (red) washing in 150 μM ranolazine. G/*V* curves were created for four distinct cells (*N* = 4), then averaged to create the composite curves shown (error bars indicate s.e.m.). Inset: example current traces.

**Fig. 2 |:**
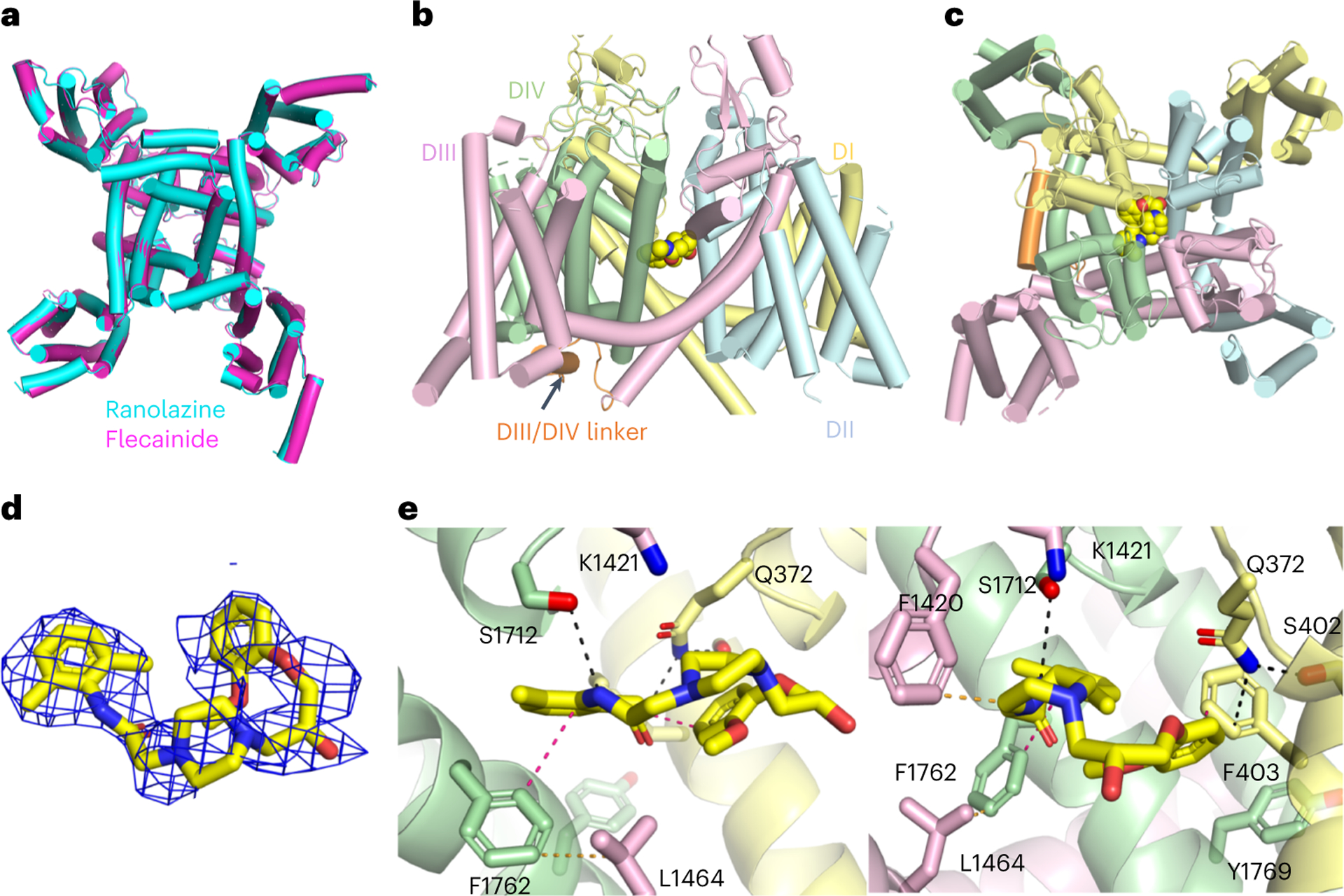
The cryo-EM structure of ranolazine bound to rNa_V_1.5c. **a**, Comparison of the structure of rNa_V_1.5c complexed with FGF12B, calmodulin and ranolazine (light blue) to that of rNa_V_1.5c complexed with FGF12B, calmodulin and flecainide (magenta). The two structures overlay nearly completely with RMSD of 0.89 Å. **b**,**c**, Orthogonal views of the complex rNa_V_1.5c with FGF12B, calmodulin and ranolazine. The channel is shown in cartoon form with each of its domains and its inactivation particle colored separately as indicated. Ranolazine is shown in spherical form with carbon purple, nitrogen blue and oxygen red. FGF12B and calmodulin are not modeled due to weak cryo-EM density. **c**, Fitting of ranolazine to the cryo-EM density map at 4*σ*. **d**, The structure of ranolazine with cryo-EM density illustrated in mesh. **e**, Orthogonal views of ranolazine in its binding site with points of interaction shown as dashed lines (pink for π-teeing, yellow for hydrophobic and black for polar), and important residues shown in stick form and labeled. The coloring of domains and residues is as indicated in **a** and **b**.

**Fig. 3 |:**
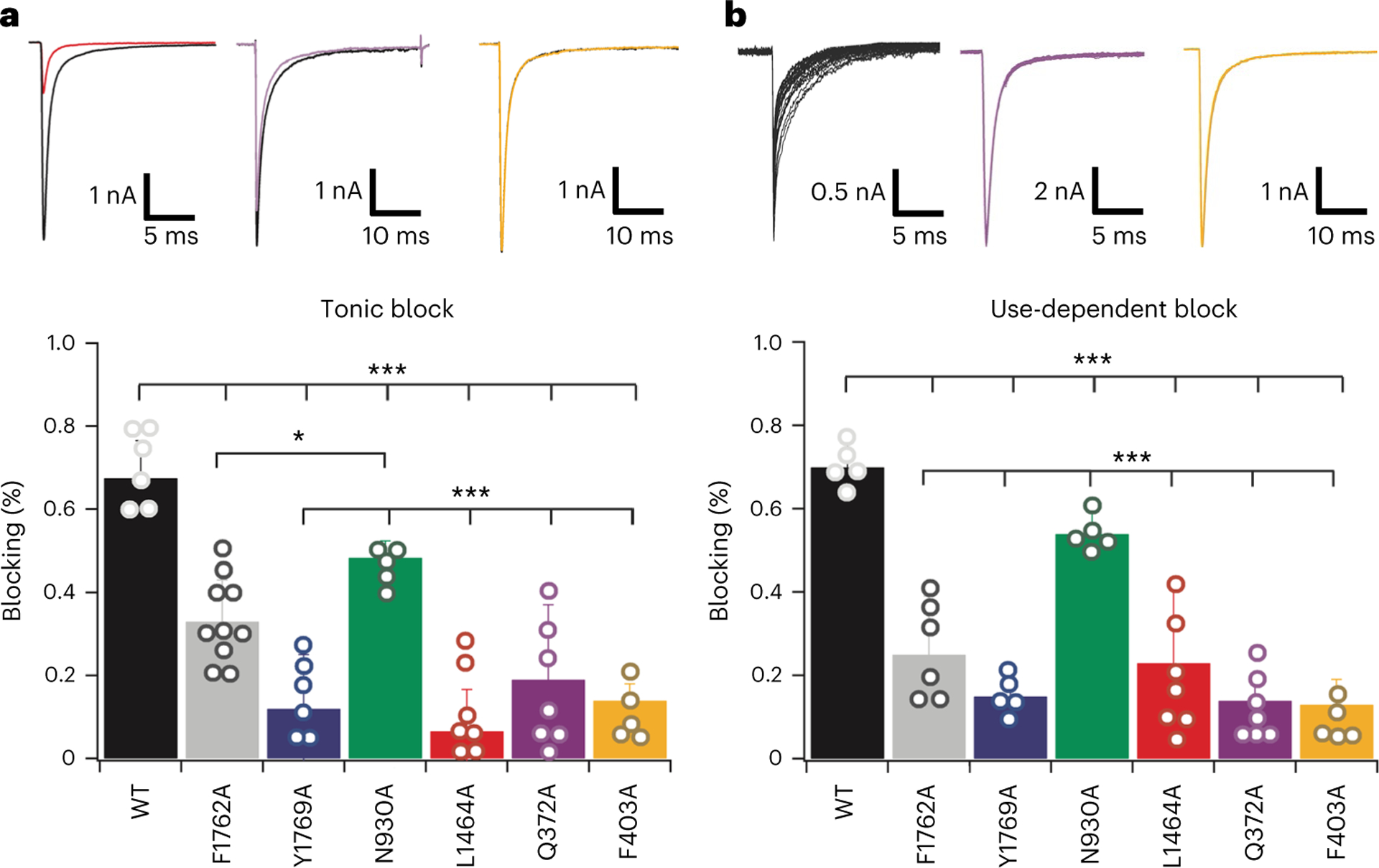
Functional effects of mutations of contact residues in the ranolazine binding site. **a**, Top: representative electrophysiological data showing tonic blockade of wild-type and selected mutants of rNa_V_1.5c as indicated. Bottom: bar graphs summarizing the effects of the indicated mutations on ranolazine blockade using 150 μM ranolazine. Number of measured cells is as follows: WT, 6 distinct cells; F1762A, 10 distinct cells; Y1769A, 6 distinct cells; N930A, 5 distinct cells; L1464A, 7 distinct cells; Q372A, 7 distinct cells; F403A, 5 distinct cells. Error bars indicate s.e.m. Two tailed *t*-test was used to check the statistical significance of mutations’ effects on ranolazine block. For WT compared with the mentioned mutants, *P* < 0.001. For N930A mutant compared with F1762A, Y1769A, L1464A, Q372A and F403A, *P* < 0.001. **b**, Effects of mutations on use-dependent block (UDB) at 1 Hz from −140 mV to 0 mV. Number of measured cells is as follows: WT, 5 distinct cells; F1762A, 6 distinct cells; Y1769A, 5 distinct cells; N930A, 5 distinct cells; L1464A, 7 distinct cells; Q372A, 7 distinct cells; F403A, 5 distinct cells. Error bars indicate s.e.m. Two tailed *t*-test was used to check the statistical significance of mutations’ effects on ranolazine block. For WT compared with the mentioned mutants, *P* < 0.001. For N930A mutant compared with Y1769A, L1464A, Q372A and F403A, *P* < 0.001.

**Fig. 4 |:**
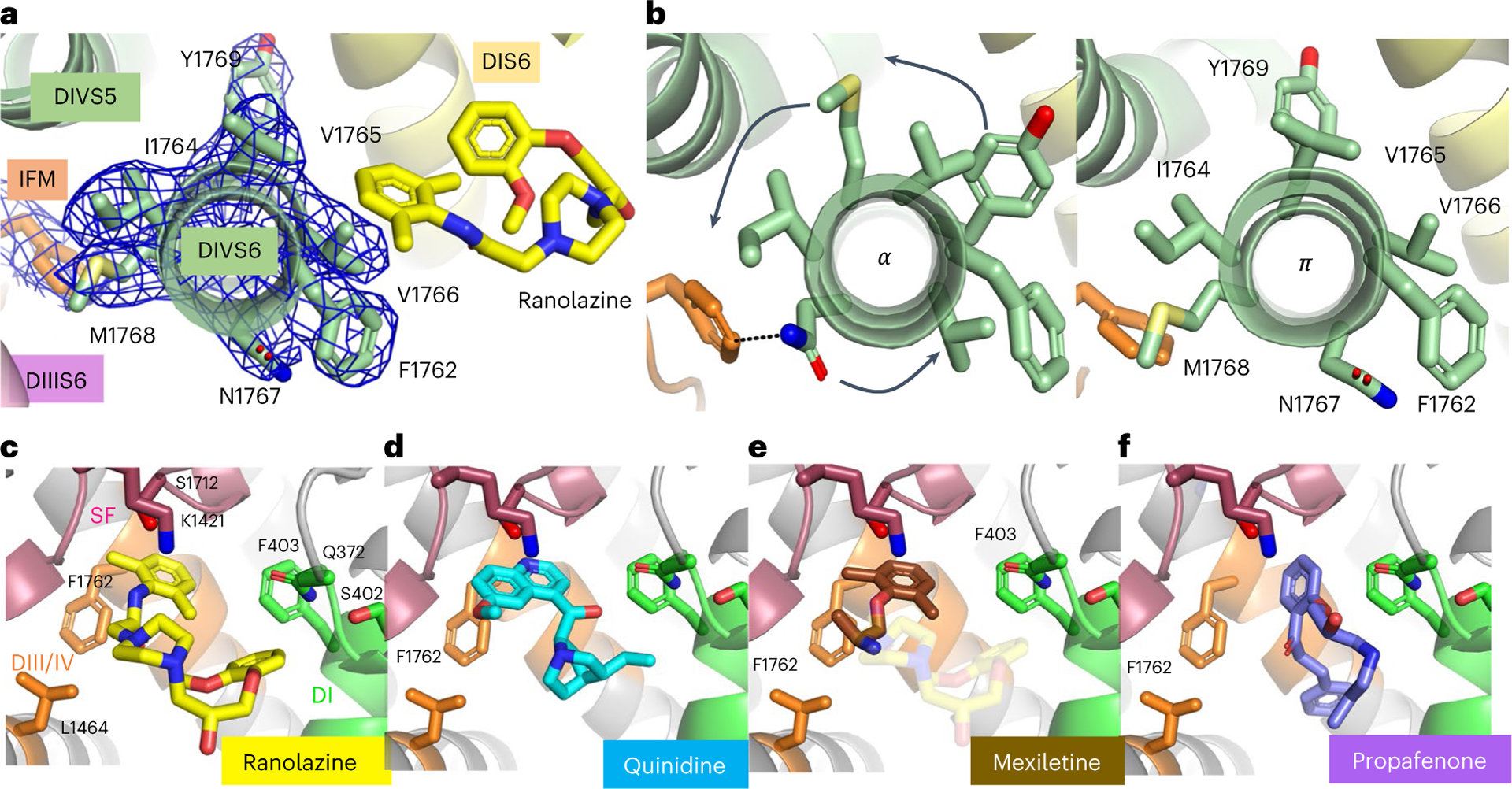
The structures of ranolazine and other AADs bound to α and π conformations of transmembrane segment DIV-S6. **a**, Structural model of DIV-S6 from the position of S1761, looking down the axis of DIV-S6. Side chains are shown as sticks for DIV-S6, the nearby IFM motif and ranolazine, highlighting the change in register that results from the π-helix conformation. Cartoon helices are shown for DI-S6, DIV-S5 and DIV-S6, and cryo-EM density is shown as blue mesh at 5*σ*. **b**, Comparison between α (left) and π (right) conformations of DIV-S6, with arrows showing the change in positions N1767, M1768 and Y1769. A dashed black line illustrates the polar interaction between N1767 and the IFM motif in the α conformation, which is replaced by a tighter, hydrophobic interaction with M1768 in the π conformation. **c**, The features of the ranolazine binding site are shown, with selectivity filter (raspberry), DIII/DIV AAD site (orange) and DI site (green). Important residues are shown in sticks and labeled. Ranolazine is shown as in Fig. 2. **d**–**f**, The binding sites of other classes of AADs are shown for comparison with ranolazine: class IA AAD quinidine, blue (**d**); class IB AAD mexiletine, brown (**e**); class IC AAD propafenone, purple (**f**). Mexiletine has been manually placed in the model shown based on the identity of its chemical structure with a portion of ranolazine (shown as transparent sticks). The structure of mexiletine in complex with Na_V_1.5 has not been determined experimentally.

## Data Availability

All data supporting the finding in this study are included in the main article and associated files. Structural data are available from the Protein Data Bank (PDB) under EMDB entry ID EMD-28887 and PDB entries ID 8F6P & 6UZ3. Source data are provided with this paper.
